# Metabolic Switch and Cytotoxic Effect of Metformin on Burkitt Lymphoma

**DOI:** 10.3389/fonc.2021.661102

**Published:** 2021-09-02

**Authors:** Irene Bagaloni, Axel Visani, Sara Biagiotti, Annamaria Ruzzo, Mohsen Navari, Maryam Etebari, Lucia Mundo, Massimo Granai, Stefano Lazzi, Alessandro Isidori, Federica Loscocco, Jiejin Li, Lorenzo Leoncini, Giuseppe Visani, Mauro Magnani, Pier Paolo Piccaluga

**Affiliations:** ^1^Department of Biomolecular Sciences (DISB), University of Urbino, Urbino, Italy; ^2^Department of Experimental, Diagnostic, and Specialty Medicine, Bologna University School of Medicine, Bologna, Italy; ^3^Department of Medical Biotechnology, School of Paramedical Sciences, Torbat Heydariyeh University of Medical Sciences, Torbat Heydariyeh, Iran; ^4^Research Center of Advanced Technologies in Medicine, Torbat Heydariyeh University of Medical Sciences, Torbat Heydariyeh, Iran; ^5^Bioinformatics Research Group, Mashhad University of Medical Sciences, Mashhad, Iran; ^6^Department of Medical Genetics, Faculty of Medicine, Mashhad University of Medical Sciences, Mashhad, Iran; ^7^Section of Pathology, Department of Medical Biotechnology, University of Siena, Siena, Italy; ^8^Health Research Institute, University of Limerick, Limerick, Ireland; ^9^Department of Pathology, Tubingen University, Tubingen, Germany; ^10^Hematology and Transplant Center, AORMN Marche Nord, Pesaro, Italy; ^11^School of Biological and Chemical Sciences, Queen Mary University of London, London, United Kingdom; ^12^Euro-Mediterranean Institute of Science and Technology (IEMEST), Palermo, Italy; ^13^School of Health, Department of Pathology, Jomo Kenyatta University of Agriculture and Technology, Nairobi, Kenya

**Keywords:** glucose metabolism, glycolysis, metformin, anaerobic glycolysis, Burkitt lymphoma

## Abstract

Altered cellular energetic metabolism has recently emerged as important feature of neoplastic cells. Indeed, interfering with cancer cell metabolism might represent a suitable therapeutic strategy. In this study, we aimed to assess glucose metabolism activation in human lymphomas and evaluate how metformin can exert its action on lymphoma cells. We studied a large series of human lymphomas (N = 252) and an *in vitro* model of Burkitt lymphoma (BL) cells. We combined molecular biology techniques, including global gene expression profiling (GEP) analysis, quantitative PCR (qPCR) and Western blotting, and biochemical assays, aimed to assess pentose phosphate pathway, tricarboxylic acid (TCA) cycle, and aerobic glycolysis rates. We found that glucose metabolism is overall enhanced in most lymphoma subtypes, based on gene expression profiling (GEP), with general shift to aerobic glycolysis. By contrast, normal B cells only showed an overall increase in glucose usage during germinal center transition. Interestingly, not only highly proliferating aggressive lymphomas but also indolent ones, like marginal zone lymphomas, showed the phenomenon. Consistently, genes involved in glycolysis were confirmed to be overexpressed in BL cells by qPCR. Biochemical assays showed that while aerobic glycolysis is increased, TCA cycle is reduced. Finally, we showed that metformin can induce cell death in BL cells by stressing cellular metabolism through the induction of GLUT1, PKM2, and LDHA. In conclusion, we unveiled glucose metabolism abnormalities in human lymphomas and characterized the mechanism of action of metformin in Burkitt lymphoma model.

## Introduction

Altered cellular energetic metabolism is emerging as one of the most important hallmarks of malignantly transformed cells. Tumor cells are subjected to several changes in metabolic pathways, among which the shift from oxidative phosphorylation to lactate fermentation, known as Warburg effect, plays a key role. In fact, normal cells convert glucose into carbonic anhydride under aerobic conditions through oxidative phosphorylation. On the contrary, cancer cells mainly produce lactate, even in the presence of sufficient levels of oxygen (1). Although aerobic glycolysis is less efficient in producing energy (2 ATP molecules instead of 36 from complete glucose oxidation), this apparent waste of glucose really constitutes a survival advantage in active proliferating cells because it makes them insensitive to transient or permanent hypoxic conditions. Moreover, lactate is not just a waste product of the process; actually, it promotes tumor invasion supporting cell migration, angiogenesis, immune escape, and radioresistance (2). The Warburg effect is still extremely relevant in the cancer research field because understanding the complex cancer energy metabolism will help to develop new approaches in early diagnosis and cancer therapy.

The biguanide metformin is the first-line therapy for type 2 diabetes (3). Retrospective studies of type 2 diabetes patients treated with metformin show a strong correlation between drug intake and reduced tumor incidence or reduced cancer-related deaths (4, 5). These findings supported the rationale of designing clinical trials using metformin as an adjuvant in chemotherapy for cancer patients (6, 7). Even if the mechanism of action of metformin is still partially unknown, and sometimes controversial, to date, two major effects of metformin have been described. The first is the inhibition of mitochondrial electron transport chain complex I (8). In fact, it has been described that metformin fuels glycolysis by impairing mitochondrial respiration through complex I inhibition, as a compensation mechanism. This limits the flux of glucose into the tricarboxylic acid (TCA) cycle that, in turn, limits acetyl-CoA levels necessary for lipid biosynthesis. Consequently, the highest concentration of pyruvate resulting from enhanced aerobic glycolysis is converted to lactate through lactate fermentation (9, 10). The second effect of metformin is the activation of AMP-activated protein kinase (AMPK), a key energy sensor in cells, which is able to switch cells from an anabolic/energy-consuming to a catabolic/energy-producing state, mimicking a condition of caloric restriction (11). Through a direct and AMPK-dependent activation of mammalian target of rapamycin (m-TOR), metformin can also induce a decrease in protein synthesis. Of note, the metabolic switch was also detected in AMPK-deficient cells indicating that the antiproliferative effect of metformin does not depend exclusively on AMPK. In fact, several AMPK-independent mechanisms have been described, underlining the pleiotropic effect of metformin in cancer (11). This study reports that metformin can exert its antineoplastic activity without activation of AMPK but rather by inhibiting downstream pathways, for example by preventing AKT phosphorylation, as reported in lung and breast cancers. In recent years, new effects of metformin, upstream AMPK activation, have been identified. For example, cell culture assay and molecular modeling studies indicated that metformin impairs, directly, glucose metabolism by inhibiting hexokinase II, the most expressed hexokinase in cancer cells, leading to its dissociation from mitochondria and activation of apoptotic signaling (11–13).

Clinical trials involving metformin as a therapy option for tumors are focusing predominantly on solid tumors such as breast, prostate, lung, and colon cancer. On the contrary, there are few evidence on the role of metformin in hematological malignancies (11). Scotland and colleagues reported that metformin elicits a reprogramming of intermediary metabolism leading to the inhibition of cell proliferation in leukemia cells (14). Recently, it has been reported that metformin induces autophagy and G0/G1 cell cycle arrest in multiple myeloma (15) and produces significant reduction in cell viability with associated alterations in oxidative phosphorylation and glycolysis in lymphoma cell lines (16).

Burkitt lymphoma (BL) is a rare, aggressive subtype of non-Hodgkin lymphoma. Despite the improvements in the diagnosis and management of the disease, treatment-related toxicity and limited therapeutic options for relapses remain a challenge in managing BL (17). Of note, although some particular aspects regarding BL metabolism have been addressed (18, 19), our overall knowledge is still very limited in this regard.

In this study, we attempt to highlight the metabolic switch induced by malignancy in B-cell-derived tumors cells and further elucidate the influence of metformin on cellular energetic metabolism in Burkitt lymphoma by performing *in vivo* gene expression profiles and *in vitro* biochemical and biomolecular analyses.

## Materials and Methods

### Case Selection

Two hundred fifty-two B-cell-derived malignancies and 25 samples of normal B-cell subpopulations were studied by gene expression profiling (GEP) analysis, including mantle cell lymphoma (MCL), chronic lymphocytic leukemia (CLL), follicular lymphoma (FL), diffuse large B-cell lymphoma, not otherwise specified (DLBCL/NOS), primary mediastinal large B-cell lymphoma (PMBCL), Burkitt lymphoma (BL), splenic marginal zone lymphoma (SMZL), plasma cell myeloma (PCM), classical Hodgkin lymphoma (cHL), and lymphocyte-predominant Hodgkin lymphoma (NLPHL) and also normal B-cell subpopulations including centroblasts (CB), centrocytes (CC), naive (N), memory cells (M), and plasma cells (PC) (20–23). Akata, Awia, Eli, Mutu, and DAUDI cell lines were also studied to verify their similarity to primary BL samples (24, 25). For more details, refer to **Supplementary Material**.

### Gene Expression Profiles Analysis

For proper comparison with our samples, gene expression values were adequately normalized (**Supplementary Figure 1**) and analyzed, as described (26–28). Briefly, the data were robust multiarray averaging (RMA) normalized and log2 transformed (29), and batch effect was removed using ComBat (30). Unsupervised [principal component analysis (PCA) and hierarchical clustering] and supervised clustering was conducted on the data (26–28). To perform the supervised gene expression analysis, ANOVA or t-test, with p ≤ 0.05 and fold change ≥2 were used; Benjamini–Hockeberg method was adopted for false discovery rate (FDR) correction (23). Gene Set Enrichment Analysis (GSEA) (27) was applied to establish which Broad Institute’s gene sets were significantly represented among different categories. We ended up with eight gene sets (**Table 1**) associated with glucose metabolism. See **Supplementary Material** for more details.

**Table 1:** Gene sets used to study glucose metabolism at gene expression level.

Broad Institute Gene Set	
BIOCARTA GLYCOLYSIS PATHWAY
GO OXIDATIVE PHOSPHORYLATION
GO REGULATION OF OXIDATIVE PHOSPHORYLATION
HALLMARK GLYCOLYSIS
HALLMARK OXIDATIVE PHOSPHORYLATION
KEGG OXIDATIVE PHOSPHORYLATION
MOOTHA GLYCOLYSIS
REACTOME GLYCOLYSIS

### Cell Line Culture, Treatment, and Cell Survival

DAUDI (BL; ATCC Cat. No. CCL-213), AKATA (BL; kindly provided by Prof. Giulia De Falco, Queen Mary University of London), and RAJI (BL; Sigma-Aldrich. Cat No. 85011429-1VL) cell lines were first studied to assess the possible cytotoxic effect of metformin by MTS assay (Abcam, USA), following the manufacturer’s instructions. Based on the (overall similar) results, DAUDI cells were chosen for the following experiments, having an intermediate IC50 among the three cell lines.

DAUDI cells were treated with 10 mM metformin (Sigma, St. Louis, MO, USA) for 24 h, prior to RNA and protein extractions. Cellular viability was evaluated by an automated cell counter at 24, 48, and 72 h after drug treatment. Prior to the experiments, DAUDI cells were compared for the expression of the genes involved in glucose metabolism to other BL cell lines to verify their comparability to primary BL samples (**Supplementary Figure 2**). Peripheral blood lymphocytes (PBL) obtained from three normal healthy blood donors were used as controls as described for DAUDI cells. Since our first aim was to unveil the effects of metformin on primary human non-neoplastic lymphocytes, we chose PBL rather than primary germinal center cells since the isolation of the latter would by itself largely affect the metabolic profile. Furthermore, based on the initial GEP, aerobic glycolysis characterized the tumor and none of the normal B-cell subsets.

More details are provided in the **Supplementary Material**.

### Pentose Phosphate Pathway, TCA Cycle, and Aerobic Glycolysis Rate Measurement

DAUDI and PBL cells treated or non-treated with metformin were subjected to rate determination of pentose phosphate pathway (PPP) and tricarboxylic acid cycle (TCA) rates using isotope tracer analyses ([1-C^14^]-glucose and [6-C^14^4]-glucose), as described (31). Aerobic glycolysis rate was calculated based on lactate production. For more details, refer to the **Supplementary Material**.

### Gene Expression Analysis by RT-qPCR

After 24 h of treatment with 10 mM metformin, total RNA was extracted from DAUDI cells and PBL (as control), complementary DNA (cDNA) was obtained, and reverse transcription quantitative PCR (RT-qPCR) was performed to evaluate the messenger RNA (mRNA) expression levels of six genes involved in the various branches of glucose metabolism: glucose transporter 1 (*GLUT-1*), hexokinase-1 (*HK1*), hexokinase-2 (*HK2*), pyruvate kinase M2 (*PKM2*), lactate dehydrogenase A (*LDHA*), and voltage-dependent anion channel 1 (*VDAC1*), using beta-actin (*ACTB*) as housekeeping gene for normalization. The relative mRNA expression of the above genes was calculated by 1/2^ΔCt^ method. See **Supplementary Material** for more details.

### Western Blotting

Western blots were performed on DAUDI cells and primary lymphocytes after 24 h of treatment with metformin at a concentration of 10 mM using monoclonal antibodies (mAb) anti-LDHA, anti-PKM, and anti-GLUT-1. Beta-actin mouse mAb was used as a loading control. Refer to the **Supplementary Material** for further details.

### Statistical Analysis

Statistical analyses of *in vitro* studies were performed with GraphPad Prism, version 8 (GraphPad Software, San Diego, CA, USA). Data are presented as means ± standard error or standard deviations of three or four independent experiments. Means for all data were compared by Wilcoxon test for paired samples and by Mann–Whitney U-test for unpaired samples. A *p* ≤ 0.05 was considered statistically significant.

## Results

### Glycolysis and Oxidative Phosphorylation Are Increased During Germinal Center Transition

**Figure 1:**
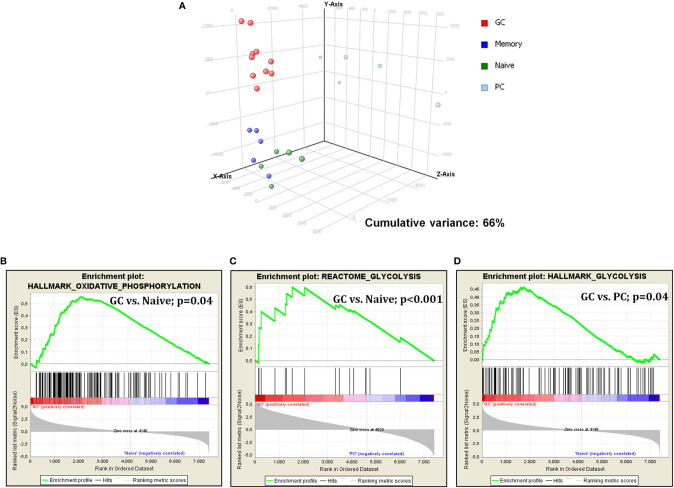
Glucose metabolism is modulated during germinal center transition. PCA on glucose metabolism associated genes showed a clear separation among normal B-cell subpopulations **(A)**. GSEA showed increased expression of genes that are involved in both glycolysis and oxidative phosphorylation in GC cells *vs*. naive cells **(B, C)** and plasma cells **(D)**.

First, we studied glucose metabolism in normal B-cell subsets. PCA, an unsupervised approach, indicated the existence of three main clusters, including GC, naive/memory (N/M), and PC, respectively. Particularly, while GC and N/M constituted two relatively homogeneous aggregates, PC samples were more dispersing, indicating a more heterogeneous gene expression pattern (**Figure 1A**). When naive and germinal center cells were compared, GC cells showed a significant enrichment in genes involved in both glycolysis and oxidative phosphorylation (**Figures 1B, C**). After germinal center transition, a reduction in glucose usage was still recorded; such reduction was particularly evident in plasma cells (**Figure 1D**), while it was not statistically significant in memory cells (data not shown).

### Glucose Metabolism Is Variably Altered in Human Malignant Lymphomas

We then investigated whether glucose-metabolism-related genes were differentially expressed in non-neoplastic B-cell populations and malignant lymphoma. Supervised analysis (t-test, p ≤ 0.05; fold change ≥2; Benjamini–Hockeberg FDR) showed that 41 genes (57 probe sets) were differentially expressed, 37 being upregulated in lymphomas and only 4 in normal cells (**Supplementary Figure 3** and **Supplementary Table 1**).

**Figure 2:**
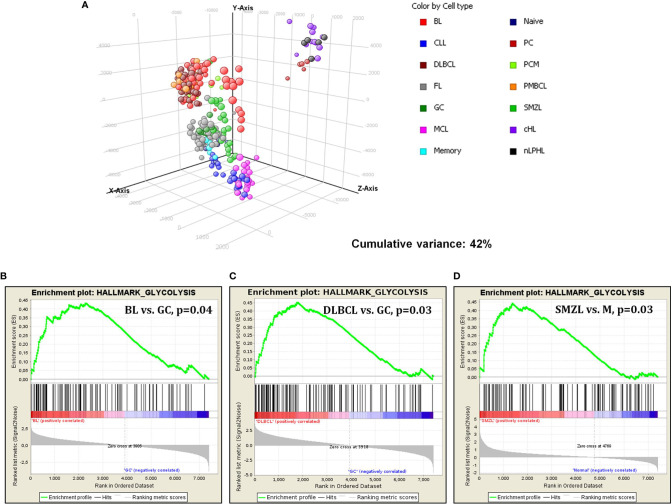
Gene expression analysis of lymphomas and normal B-cell by DNA microarrays. **(A)** PCA showed specific clustering of lymphomas and normal cells based on glucose metabolism associated genes. Each sphere represents a sample. **(B–D)** GSEA revealed significant enrichment in genes involved in glycolysis in BL, DLBCL and SMZL, when these tumors were confronted to their normal counterparts.

However, when cases were clustered by principal component analysis (PCA), it was evident that different tumor types behaved differently. In fact, we observed four main clusters including (1) aggressive GC-derived lymphomas (DLBCL/NOS, PMBCL, and BL); (2) FL, SMZL, and naive, GC, and memory B cells; (3) MCL and CLL; and (4) Hodgkin lymphomas and plasma cells (**Figure 2A**).

Subsequently, we studied glucose-metabolism-associated gene sets in the individual tumor types, comparing the neoplastic cells *via* GSEA with their normal, non-neoplastic counterparts. Burkitt lymphoma, compared to GC B cells, showed a significant enrichment in genes related to glycolysis. Of interest, no enrichment in oxidative phosphorylation was recorded, indicating a possible metabolic shift toward aerobic glycolysis (Warburg effect) (**Figure 2B**). Similarly, DLBCL cases were compared to GC cells, plasmablasts (the supposed counterpart of ABC-type DLBCL) not being available. Again, a considerable enhancement in glycolysis in favor of DLBCL cells was recorded (**Figure 2C**). Comparing SMZL to memory B cells showed enhancement in glycolysis as well (**Figure 2D**). Conversely, none of the other entities showed a significant enrichment in either glycolysis or oxidative phosphorylation when compared to the corresponding counterpart (data not shown).

Overall, it appeared that although most tumors presented with a variable augment in the expression of glucose metabolism associated genes, only few entities showed a remarkable and consistent increase in them. Particularly, BL and DLBCL turned to out to further upregulated these genes even if compared to highly proliferating GC B cells that already showed a consistent increase in glucose usage compared to nodal resting B cells in our analysis. Based on the above findings indicating a potential usefulness of drugs interfering with glucose metabolism in DLBCL, we further investigated BL.

### Gene Expression of Genes Involved in Glucose Metabolism in DAUDI Cells *Versus* PBL

**Figure 3:**
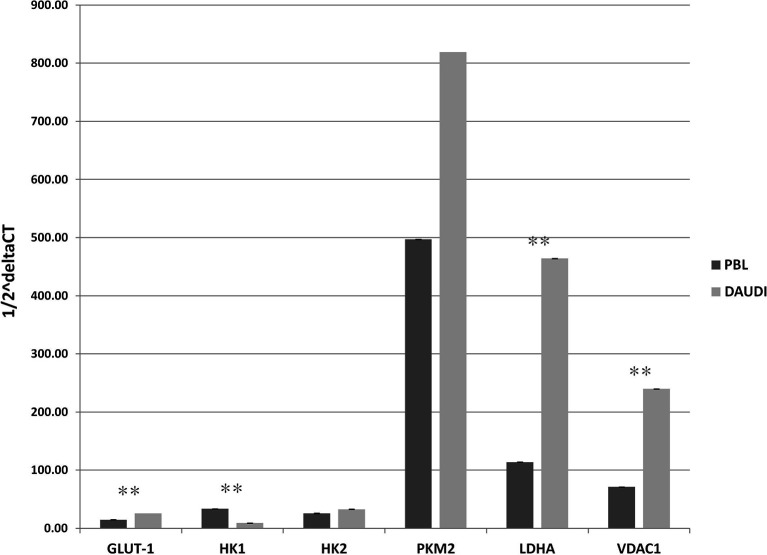
Gene expression of glucose metabolism genes in DAUDI cells *vs*. PBL. The gene expression of selected targets involved in glucose metabolism was evaluated by RT-qPCR in both DAUDI cells and PBL. Beta-actin was used as reference gene. Values represent the relative amount of each transcript in the two cell types. Values are means and SEM of four independent experiments (Mann-Whitney U-test, **two-tailed p-values ≤ 0.05).

To validate the results observed in the gene sets from microarray data, which reported the upregulation of genes involved in glucose metabolism in patients affected with BL, we investigated the gene expression of six selected genes involved in glucose metabolism in both DAUDI and PBL by means of RT-qPCR. As expected, we found that the main genes involved in Warburg effect were upregulated in BL-originated DAUDI cells compared to healthy lymphocytes. Particularly, a statistically significant upregulation was observed as far as *GLUT-1*, *LDHA*, and *VDAC1* (**Figure 3**). Moreover, *HK1* was found to be downregulated in DAUDI cells with respect to PBL, while *HK2* was found to be upregulated. This may be due to the fact that *HK2*, instead of *HK1*, is frequently upregulated in cancer, and, in addition, the binding of *HK2* to the external mitochondrial membrane in a complex with *VDAC1* is known to contribute to fuel the glycolytic process (32).

### Overall Glucose Metabolism in DAUDI Cells

**Figure 4:**
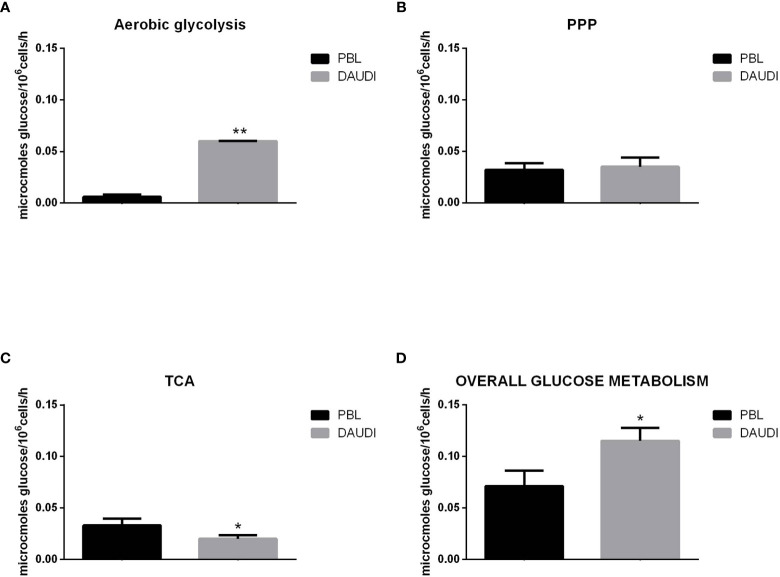
Glucose metabolism in DAUDI cells *vs*. PBL. Values are means and SEM of four independent experiments (Wilcoxon signed rank test, two-tailed; *p-values ≤ 0.05; **p-values < 0.005). **(A)** Bars represent the rates of glucose consumption in aerobic glycolysis leading to lactate production. Effective rates of utilization of radiolabeled glucose in PPP **(B)** and TCA (Krebs) cycle **(C)** was evaluated by administration of [1-C^14^]-glucose or [6-C^14^]-glucose and subsequent monitoring of the emitted ^14^CO_2_, which is directly proportional to the consumed glucose. PPP rates were determined as the difference between the rates of radiolabeled carbon dioxide production from [1-C^14^]- and [6-C^14^]-glucose. TCA cycle rates were determined by monitoring the production of radiolabeled carbon dioxide only produced by [6-C^14^]-glucose. Overall glucose consumption **(D)** was calculated as the sum of glucose consumption in aerobic glycolysis, PPP and TCA cycle.

To further validate the results obtained by gene expression assays, we assessed the *in vitro* overall glucose consumption, by focusing on the rate of the PPP, TCA cycle, and aerobic glycolysis branches, in cellular model of BL (DAUDI cells) and normal PBL as control. As expected, we demonstrated that overall glucose metabolism was increased in DAUDI cells with respect to PBL of about twofold because of tumor metabolism switch, being the main effects on aerobic glycolysis (increased) and mitochondrial metabolism (decreased) (**Figure 4**).

### Effect of Metformin on Cell Viability

To test the possibility of treatment with metformin, we first assessed the cytotoxic effect of the drug on AKATA, DAUDI, and RAJI cells by MTS assay and assess the IC50 after 48 h of exposure. We observed a relatively similar dose-dependent cytotoxic effect. Particularly, the IC50 was 16.56, 12.82, and 9.81 mM, for the three lines, respectively (**Supplementary Figure 4**). Based on that, DAUDI (the one showing the intermediate degree of sensitivity) was used for the following experiments.

**Figure 5:**
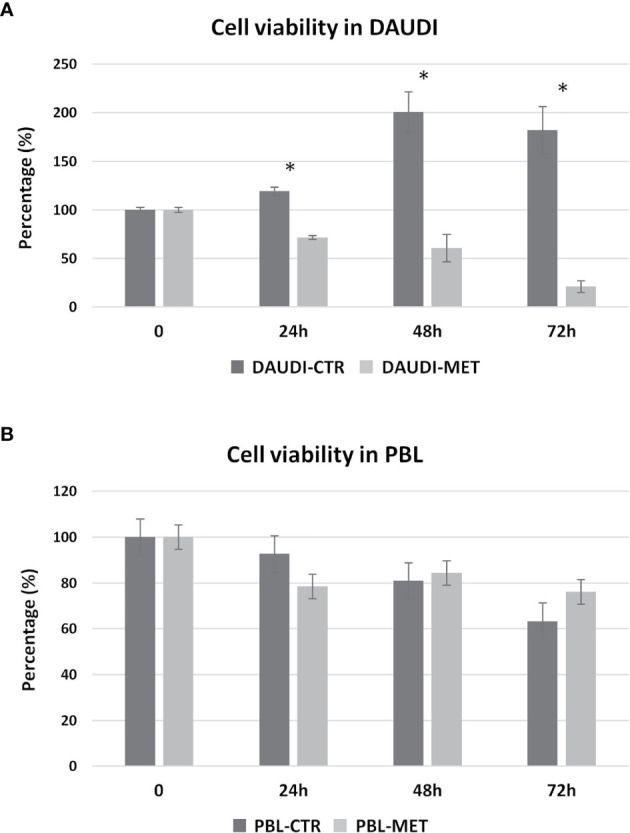
Metformin effect on cell viability. DAUDI cells **(A)** and PBL **(B)** were treated with 10 mM metformin for 24, 48 and 72 hours. CTR, untreated cells; MET, treated cells with 10mM Metformin. Significance is expressed by comparing treated cells *versus* the respective untreated ones (Wilcoxon signed rank test; *two-tailed p-values ≤ 0.05).

We then further studied the cytotoxic effects of metformin on DAUDI cells, treating in parallel PBL as control. To this end, cells were treated with 10 mM metformin for 24, 48, and 72 h. DAUDI cells showed a strong and significant reduction in cell viability after 24 h of treatment, and this effect increased at longer periods of treatment (**Figure 5A**). On the contrary, metformin treatment slightly affected healthy lymphocytes (**Figure 5B**). In fact, the observed reduction in cell viability could mainly be attributable to the physiological death of primary lymphocytes, which were not supplemented with specific growth factors to avoid any interference in metabolism assays. Since the IC50% in DAUDI cells was almost reached after 24 h of treatment, all following assays were performed under this condition.

### Effect of Metformin on Glucose Metabolism

**Figure 6:**
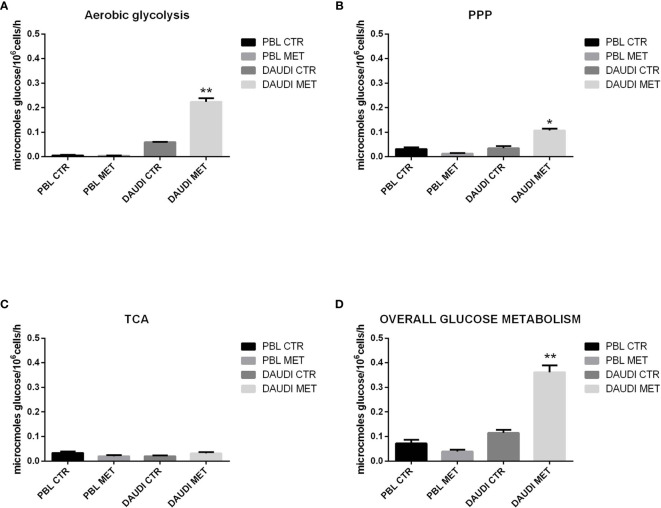
Effect of metformin on glucose metabolism in DAUDI and PBL cells. Panel **(A)** represent the rates of glucose consumption in aerobic glycolysis producing lactate, while panel **(B, C)** show the rate of PPP and TCA. Effective rates of utilization of radiolabeled glucose in PPP and TCA cycle (Krebs) was confirmed by administration of [1-C^14^]-glucose and [6-C^14^]-glucose and subsequent monitoring of emitted ^14^CO_2_, which is directly proportional to the glucose consumed. PPP rates were determined as the difference between the rates of radiolabeled carbon dioxide production from [1-C^14^]- and [6-C^14^]-glucose. TCA cycle rates were determined monitoring the production of radiolabeled carbon dioxide only produced by [6-C^14^]-glucose. Overall glucose consumption **(D)** was calculated as the sum of glucose consumption in aerobic glycolysis, PPP and TCA cycle. CTR: untreated cells; MET: treated cells with 10mM metformin. Values are means and SEM of three independent experiments (Wilcoxon signed rank test, two-tailed**;** *p-values ≤ 0.05; **p-values ≤ 0.005).

To test if the cytotoxic effect of metformin was somehow due to changes in the overall energetic and metabolic status, we assessed glucose metabolism by focusing on the rate of aerobic glycolysis, PPP, and TCA in DAUDI cells and PBL treated or untreated with metformin (**Figure 6**).

As a first point, we focused on the aerobic glycolysis rate by measuring the amount of lactate produced. As shown in **Figure 6A**, we found that lactate production was significantly increased after metformin treatment. We next focused on the PPP and TCA rates. The results showed that, in DAUDI cells, the amount of glucose utilized in the oxidative decarboxylation of the hexose monophosphate pathway increased significantly after metformin administration, consistent with an increased rate of the PPP (**Figure 6B**). On the other hand, the amount of glucose utilized in the oxidative decarboxylation during TCA cycle was slightly affected by metformin treatment (**Figure 6C**).

The overall glucose metabolism, calculated as the sum of PPP, TCA, and aerobic glycolysis, indicated more than 2-fold upregulation in DAUDI cells after metformin treatment (**Figure 6D**). On the contrary, in PBL, the overall glucose metabolism was slightly downregulated after metformin treatment, since it utilizes less glucose in both PPP and TCA cycle, while aerobic glycolysis remained unchanged. As for cell viability, this could be explained by the physiological resting of lymphocytes, which were not supplemented with specific growth factors.

### Effect of Metformin on Gene Expression and Protein Levels

**Figure 7:**
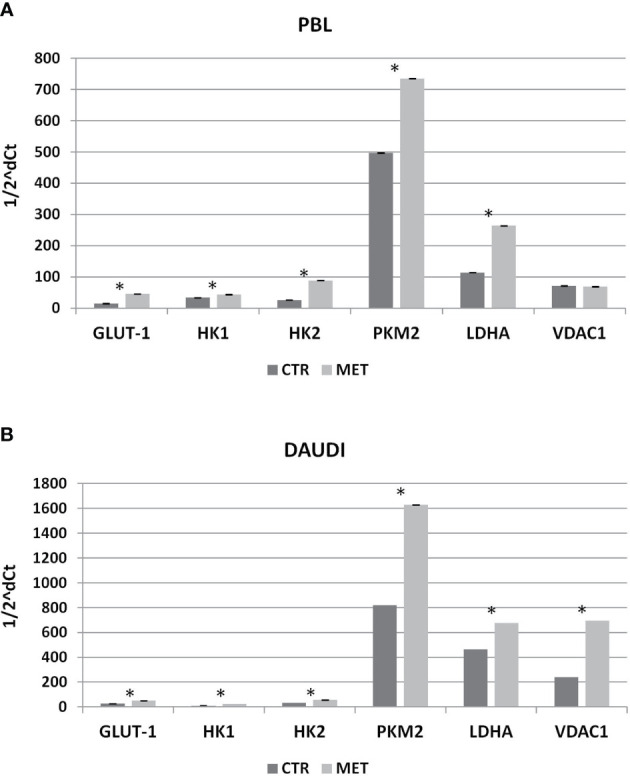
Effect of Metformin on gene expression profile of PBL **(A)** and DAUDI cells **(B)**. The effect of metformin on gene expression of selected targets involved in Warburg’s effect was evaluated by RT-qPCR after 24 hours of 10 mM metformin treatment in both DAUDI cells and PBL. Beta-actin was used as the reference genes. Values represent fold changes of each target with respect to the untreated control (Wilcoxon matched-pairs signed rank test; *p-values ≤ 0.05).

Subsequently, we investigated whether this metabolic reprogramming could be the result of rearrangement in the gene expression profile. To this end, we selected a panel of genes involved in the Warburg effect including *HK1*, *HK2*, *PKM2*, *VDAC1*, *GLUT-1*, and *LDHA*, and mRNA levels were analyzed by RT-qPCR. In **Figure 7**, the relative expression levels, calculated by means of the 1/2^ΔCt^ method, are shown (**Figure 7A**, PBL; **Figure 7B**, DAUDI).

In DAUDI cells, we found upregulation of the main genes involved in glucose metabolism, the main effect being observed on *GLUT-1*, *HK1*, *HK2*, *PMK2*, *LDHA*, and *VDAC1*. On the other hand, in PBL, the main effect was on *GLUT-1*, *HK1*, *HK2*, *PKM2*, and *LDHA* genes. As final point, we evaluated whether the observed increase in gene expression was supported by a corresponding increase in protein level expression. As shown in **Supplementary Figure 5**, in DAUDI cells treated with metformin, we demonstrated a corresponding increase in GLUT-1, PKM2, and LDHA protein levels. Regarding PBL, the upregulation at mRNA level does not correspond to an increase at protein level, consistent with the lack of metformin effect observed at metabolic level.

## Discussion

Multiple molecular mechanisms, both intrinsic and extrinsic, converge to alter core cellular metabolism and to provide support for the three basic needs of dividing cells: rapid ATP generation to maintain the energy status, increased biosynthesis of macromolecules, and the strict maintenance of an appropriate redox status. To meet these needs, cancer cells acquire alterations to the metabolism of all four major classes of macromolecules: carbohydrates, proteins, lipids, and nucleic acids (33). In this study, we explored for the first time glucose metabolism in a large panel of malignant lymphomas and normal B-cell subsets. Although the tumors, taken together, showed a slight, but still significant (p = 0.05), enrichment in the expression of glycolysis associated genes, later only few tumor types (DLBCL, SMZL, and BL) demonstrated a significant enrichment when compared to the corresponding normal counterparts. Indeed, very little is currently known concerning SMZL metabolism, a fact that certainly warrants further exploration. In fact, differently from DLBCL and BL, the proliferation rate, usually low in MZL, cannot, as obviously as in more aggressive lymphomas, be regarded as the driving factor for glycolysis increase. On the other hand, DLBCL is already known to be characterized, generally speaking, by “increased glucose metabolism” based on increased glucose uptake documented at [18F]-fluorodeoxyglucose positron emission tomography/computed tomography ([18F]Ł-FDG-PET/CT), this being related to tumor proliferation (34). Furthermore, the activation of the phosphoinositide 3-kinase (PI3K)/Akt/mTOR pathway was shown to be able to promote this metabolic change rendering DLBCL cells glucose dependent (35). As a consequence, very recently, it was shown that glyceraldehyde 3-phosphate dehydrogenase (GAPDH) expression could predict not only the metabolic status but also response to chemotherapy and specific metabolism-interfering therapy in DLBCL patients (36). As far as BL is regarded, it is known that MYC oncogene, the molecular hallmark of the tumor, is responsible for profound effects on cell metabolism (37). However, only very little was specifically reported in BL, mainly concerning a possible positive effect of metformin when BL cells are treated with selected apoptosis and signaling inhibitors (16). Based on such preliminary evidence and the rational of inhibiting pathways downstream the driving oncogene MYC, we further investigated this tumor and served as a model for this study. Interestingly, different from this previous study, we found that lymphoma cells preferred aerobic glycolysis, consistent with the Warburg effect, rather than oxidative phosphorylation. Furthermore, we also showed gene expression in a large set of primary cases, while Chukkapalli and colleagues mainly referred to cell lines (16).

To further validate data from gene expression microarrays, we performed analyses by qRT-PCR on six target genes involved in glucose metabolism in DAUDI BL cells and PBL. Unfortunately, primary lymphoma cells were not considered suitable for validation analyses, since functional studies would have been significantly affected by tissue manipulation and cells isolation. *GLUT-1*, *LDHA*, and *VDAC1* were significantly upregulated in DAUDI cells with respect to PBL; on the contrary, *HK1* was significantly downregulated. Upregulation of *GLUT-1* and *LDHA* is consistent with the enrichment of glycolysis gene set and suggested that lymphoma cells, as for most tumors, undergo the Warburg effect. *HK1* is defined as a mitochondrial hexokinase because of its interaction with *VDAC1*, which is in the outer mitochondrial membrane (OMM), and regulates ATP flux from the mitochondria to cytoplasm. The attachment to the OMM promotes the coupling of glycolysis to mitochondrial oxidative phosphorylation (38). Therefore, *HK1* downregulation is possibly correlated with the impairment of respiratory activity. Tseng and colleagues (39) reported that *HK1* silencing induces a switch in energy metabolism specifically from oxidative phosphorylation to aerobic glycolysis and enhances tumor malignancy, increasing cancer cell proliferation and metastasis. The upregulation of *VDAC1* in DAUDI cells is probably due to a compensation mechanism, which reflects the increased requirement of energy consequent to the active proliferative state of cancer cells. Subsequently, we assessed glucose metabolism and the specific rate of the pentose phosphate pathway (PPP) and the citric acid cycle (TCA) by isotope tracer analyses in DAUDI cells and PBL. Moreover, we measured lactate production as an indicator of aerobic glycolysis rate. As expected, we demonstrated that overall glucose metabolism was increased about twofold in DAUDI cells when compared with PBL. This increase was mainly due to the induction of aerobic glycolysis. These data confirmed gene expression profiles and showed that tumorigenesis *per se* induces a shift from an anabolic state to a catabolic state in BL. In the light of the current state of research in the field, we investigated the effects of metformin on the viability and cellular metabolism in BL. We found that metformin strongly affected viability in DAUDI cells, while PBLs were not affected by the treatment, indicating the ability of metformin to selectively target cancer cells. These observations suggested a possible therapeutic role for targeting cellular metabolism in BL and gave the rationale to further explore the mechanism of action of metformin. Here, we demonstrate that metformin exerts its major effect on metabolic pathways, enhancing even more the rate of aerobic glycolysis that, pushed to the limit, probably leads cancer cells to death. It should be noted, however, that the IC50 that we observed for metformin in BL cell lines is relatively high (in the order of magnitude of mM). Despite being in line with previous studies on lymphomas, this might certainly affect the actual feasibility of *in vivo* treatments. On the other hand, the association of lower doses to chemotherapy or targeted agents might certainly maintain a biological and therapeutic effect, which need further investigation *in vivo*.

To determine the overall energetic and metabolic changes induced by the drug, we assessed the overall glucose metabolism by evaluating the rate of glucose consumption both through TCA and PPP in DAUDI cells treated or untreated with metformin. Metformin was chosen as already available for clinical use and safely administrable during chemotherapy and obviously interfering with glucose metabolism. We performed isotope tracer analyses, which revealed that the amount of glucose consumed in the oxidative decarboxylation of the hexose monophosphate pathway increased after metformin administration, consistent with an increased rate of the PPP. On the other hand, the amount of glucose employed in oxidative decarboxylation during TCA cycle was slightly affected by metformin treatment. Moreover, metformin induces a strong increase in lactate production in DAUDI cells due to a rise in aerobic glycolysis rate, suggesting that pyruvate is preferentially converted to lactate as opposed to entering the TCA cycle. Taken together, our data indicate that metformin treatment induces a strong increase in the overall glucose consumption and that, instead of mitochondrial metabolism, glycolysis intermediates are probably used to fuel aerobic glycolysis and the PPP, which is one of the most important pathways for NADPH regeneration that is required for both the nucleotide synthesis and cell survival under stress conditions. Under normal conditions, 90% of glucose is directed to glycolysis, while only 10% is directed to PPP, while under oxidative stress conditions, the rate of PPP can be dramatically increased to promote antioxidant defense (40, 41). In our conditions, we found that about 30% of total glucose was shunted in the PPP, while the remaining (about 60%) was directed to aerobic glycolysis. Aerobic glycolysis is potentiated probably as a compensation mechanism because inhibition of complex-I impairs ATP production and prevents NADH oxidation through oxidative phosphorylation, thus requiring oxidation of cytosolic NADH by the conversion of pyruvate to lactate (10). These results are consistent with the observations of Scotland et al. (14) reporting that, in leukemic cells, metformin induces an increase in glucose utilization through both the aerobic glycolysis (Warburg effect) and pentose phosphate pathway as a compensatory effect of its early inhibition of mitochondrial energetics. On the contrary, PBL consumed less glucose in both PPP and TCA cycle after metformin treatment, while the lactate production was not affected. This could be explained by the physiological resting of lymphocytes, which were not supplemented with specific growth factors. Finally, we investigated metformin effects on gene expression and protein levels of the six candidate genes, as already mentioned. We found that, at mRNA level, metformin induces a strong upregulation of the overall targets in DAUDI cells; the main effect was observed on *GLUT-1*, *HK1*, *HK2*, *PKM2*, *LDHA*, and *VDAC1*, consistent with the increased glucose uptake and the increased rate of aerobic glycolysis. Upregulation of GLUT-1, PKM2, and LDHA was also confirmed at protein level in DAUDI cells. About PBL, the main effect was on *GLUT-1*, *HK1*, *HK2*, *PKM2*, and *LDHA* genes; however, these changes in gene expression do not reflect an increase in protein levels and metabolic utilization in the respective metabolic pathways. Nonetheless, off-targets effects of metformin accounting for its cytotoxicity at higher doses cannot be excluded. In conclusion, our data showed for the first time that (1) glucose metabolism is overall disrupted in human malignant lymphomas, (2) Burkitt lymphoma presents with a specific metabolic shift to aerobic glycolysis, and (3) metformin can kill BL cells by determining a strong variation in glucose usage. These data support further investigation on the possible use of metformin in Burkitt lymphoma treatment as an alternate and/or in combination with approved treatments. Based on our results, clinical trials aimed to interfere with lymphoma glucose metabolisms are indicated, while further biochemical and metabolomic studies are warranted to shed light on the specific features of other lymphoma subtypes.

## Data Availability Statement

The original contributions presented in the study are included in the article/**Supplementary Material**. Further inquiries can be directed to the corresponding authors.

## Author Contributions

PP, GV, and MM conceptualized and designed the study, supervised the analyses, and critically revised the manuscript. MN, AV, and ME were responsible for gene expression profiling analysis. IB, SB, AR, AI, and FL were responsible for biochemical analysis. LM, MG, SL, and LL contributed to cell culturing and viability assays with metformin. JL contributed to manuscript writing and critically revised it. All authors contributed to the article and approved the submitted version.

## Funding

This work was supported by AIL-Pesaro Onlus (2019, AV), BolognAIL (2020, PP), RFO DIMES (2018, PP), and FIRB Futura 2011 RBFR12D1CB (PP). IB is an AIL-Pesaro Onlus fellow.

## Conflict of Interest

The authors declare that the research was conducted in the absence of any commercial or financial relationships that could be construed as a potential conflict of interest.

## Publisher’s Note

All claims expressed in this article are solely those of the authors and do not necessarily represent those of their affiliated organizations, or those of the publisher, the editors and the reviewers. Any product that may be evaluated in this article, or claim that may be made by its manufacturer, is not guaranteed or endorsed by the publisher.
