# Supplementary material for: Metabolic Switch and Cytotoxic Effect of Metformin on Burkitt Lymphoma
Source: Front Oncol. 2021 Sep 2;11:661102. doi: 10.3389/fonc.2021.661102 (PMC8454268; doi:10.3389/fonc.2021.661102)
Supplement: Supplementary file 1 [file DataSheet_1.pdf]

Supplementary Material

1 Supplementary Materials and Methods

Case selection

Two hundred fifty-two B-cell derived malignancies and twenty-five samples of normal B-cell subpopulations were studied by gene expression profiling (GEP) analysis. For all cases, GEPs were previously generated at our or other Institutions. In particular, we included mantle cell lymphoma (MCL; *GSE16455*, N=22), chronic lymphocytic leukemia (CLL; *GSE12195* and *GSE16455*, N=27), follicular lymphoma (FL; *GSE12195*, N=40), diffuse large B-cell lymphoma, not otherwise specified (DLBCL/NOS; *GSE4732*, N=46), primary mediastinal large B-cell lymphoma (PMBCL; *GSE4732*, N=20), Burkitt lymphoma (BL; *GSE4732*, N=46), splenic marginal zone lymphoma (SMZL; *GSE35426*, N=24), plasma cell myeloma (PCM; *GSE24080*, N=10), classical Hodgkin lymphoma (cHL; *GSE12453*, N=12), and lymphocyte-predominant Hodgkin lymphoma (NLPHL; *GSE12453*, N=5). Samples of normal B-cell sub-populations including centroblast (CB; N=5), centrocytes (CC; N=5), naïve (N; N=5), memory cells (M; N=5), and plasma cells (PC; N=5) have been already detailed [1-4] (*GSE12195*, N=40). Akata, Awia, Eli, Mutu and DAUDI cell lines were also studied to verify their similarity to primary BL samples (GSM2683397-2683410; GSM886973) [5, 6]. Raw gene expression data are available at GEO Database (Gene Expression Omnibus of the National Center for Biotechnology Information-NCBI, (http://www.ncbi.nlm.nih.gov/projects/geo/).

Gene Expression Profiles Analysis

For proper comparison with our samples, gene expression values were adequately normalized, as previously described (Supplementary Figure 1) [7-9]. The CEL files were quantile normalized and the values were transformed into log2 using RMA method implemented by affy Bioconductor R package [10]. The data was subjected to batch effect removal using ComBat package in R [11]. This was followed by mean-variance normalization in geWorkbench 2.4.1. The data was further analyzed using GeneSpring GX 12 (Agilent, USA). Briefly, unsupervised clustering was generated using a hierarchical algorithm based on the average-linkage method [7, 8]. The distance between two individual samples was calculated by Pearson distance [9]. Only genes displaying a twofold average change in the expression level across the whole panel were chosen to generate the hierarchical clustering. Principal component analysis (PCA) was performed as previously [7]. To perform the supervised gene expression analysis, ANOVA or T-test, with p value$\leq$0.05 and fold change$\geq$2 were used; Benjamini-Hockeberg method was adopted for false discovery rate (FDR) correction [4].

Gene Set Enrichment Analysis (GSEA) [12] with t test–based P values for weighting statistics was applied in order to establish whether specific cell functions and biological processes, classified as Broad Institute gene sets, were significantly represented among the deregulated genes. To study glucose metabolism, we selected gene sets based on their association with such process. We ended up with 8 gene sets (Table 1), including 695 probe sets corresponding to 495 unique genes.

Gene expression studies were conducted according to MIAME guidelines (https://www.ncbi.nlm.nih.gov/geo/info/MIAME.html). Raw data are already available in GEO (see above).

Cell line culture, treatment and cell survival

DAUDI cells (Burkitt; Cat. No: CCL-213) were purchased from American Type Culture Collection (ATCC) and were maintained in RPMI-1640 (Lonza, Basel, Switzerland) containing 1% glutamine (Lonza, Basel, Switzerland), 10% fetal bovine serum (FBS, LifeTechnologies, Gaithersburg, MD, USA) and 1% penicillin-streptomycin (Lonza, Basel, Switzerland). Cells were incubated at 37°C with 5% CO2 and treated with 10 mM metformin (Sigma, St. Louis, MO, USA) for 24 hours, prior to RNA and protein extractions. Cellular viability was evaluated by TC20™ Automated Cell Counter (Bio-Rad, Hercules, CA, USA) 24, 48 and 72 hours after drug treatment. Prior to experiments, DAUDI cells were evaluated at gene expression level, to verify their comparability to primary BL samples (Supplementary Figure 2). Briefly, Akata, Awi, Eli, Mutu and DAUDI Burkitt lymphoma cell lines were studied for the expression of the genes involved in glucose metabolism. All of them clustered with primary tumors, being distinct from normal B-cell subsets. This confirmed their reliability for functional studies concerning glucose metabolism. Since all cell lines appeared quite similar in this regard, DAUDI cells were chosen due to their availability in our laboratories.

Peripheral blood lymphocytes (PBL) obtained from three normal healthy blood donors were used as controls. Briefly, white blood cells were separated from red blood cells and granulocytes by separation of blood samples onto Ficoll-Paque density gradient media (Sigma, St. Louis, MO, USA). Next, monocytes were separated from lymphocytes by adhesion of suspension cells to petri dishes. The supernatant, containing the cells of interest, was then collected, cultured in completed RPMI-1640 and treated as already described for DAUDI cells.

Pentose phosphate pathway, TCA cycle and aerobic glycolysis rate measurement

DAUDI and PBL cells treated or non-treated with metformin were subjected to rate determination of pentose phosphate pathway (PPP) and tricarboxylic acid cycle (TCA) rates. In case of PPP, it was determined by incubating cells with tracer doses of [1-C^{14}]-glucose and [6-C^{14}4]-glucose. After its internalization, glucose is rapidly phosphorylated to glucose 6-phosphate (G6P) to enter glycolysis. G6P can also be shunted to PPP. In the oxidative branch of PPP, it is first oxidized to 6-phosphogluconate and then decarboxylated to ribulose 5-phosphate with the consequent emission of CO_2. Emission of CO_2 can be monitored by administration of [1-C^{14}]-glucose. G6P, which continues with glycolysis, can enter the tricarboxylic acid cycle (TCA) encountering two subsequent decarboxylations. Emissions of CO_2 during TCA can be monitored by administration of [6-C^{14}]-glucose. The PPP rate was calculated as the difference between the $^{14}CO_2$ derived from [1-C^{14}]-glucose (metabolized in both PPP and TCA) and that derived from [6-C^{14}]-glucose (metabolized only in the TCA). To this end, cells were cultured in completed RPMI containing traces of either [1-C^{14}]-glucose or [6-C^{14}]-glucose. Trapping of the radioactive $^{14}CO_2$ metabolized from cells and scintillation counting was performed as previously reported [13].

Aerobic glycolysis rate was calculated based on lactate production. DAUDI or PBL cells treated or non-treated with metformin were collected, counted for normalization and subjected to lactate

analysis using Lactate Assay Kit (Sigma, Cat.# MAK064) according to the manufacturer's instructions. Results are expressed as micromoles of glucose consumed in the aerobic pathway to produce lactate.

Gene expression analysis by RT-qPCR

After 24-hours of treatment with 10 mM metformin, total RNA was extracted from DAUDI cells and PBL (as control) using RNeasy mini kit (Qiagen, Hilden, Germany). The respective cDNA was obtained by SuperScript VILO cDNA Synthesis kit (Invitrogen, UK) according to the manufacturer's instructions. Reverse transcription quantitative PCR (RT-qPCR) was then performed to evaluate the mRNA expression levels of six genes involved in the various branches of glucose metabolism: glucose transporter 1 (GLUT-1), hexokinase-1 (HK1), hexokinase-2 (HK2), pyruvate kinase M2 (PKM2), lactate dehydrogenase A (LDHA) and voltage-dependent anion channel 1 (VDAC1). Beta-actin (ACTB) was used as housekeeping gene for normalization. RT-qPCR reactions were carried out by using TaqMan Gene Expression Assay and TaqMan Gene Expression Master Mix (Applied Biosystems, Foster City, CA, USA) on an Applied Biosystems 7500 Real-Time PCR System. The 20 µl PCR mixture consisted of 30 ng cDNA, 10 µl 2X TaqMan Gene Expression Master Mix, 1 µl of 20X TaqMan Gene Expression Assay and 5 µl of RNase and DNase-free deionized water. The reaction mixtures were incubated at 50°C for 2min, followed by 95 °C for 15 min and by 40 amplification cycles at 95 °C for 15 s, 60 °C for 1 min, as manufacturer instructions. The relative mRNA expression of each candidate gene was expressed as the $\Delta Ct = Ct(\text{target gene}) - Ct(\text{reference gene})$. In a further step, a second relative parameter was added as produced by the $1/2^{\Delta Ct}$ method. Statistical analyses were performed on the ΔCt values.

Western Blotting

Western blots were performed on DAUDI cells and primary lymphocytes after 24h treatment with metformin at a concentration of 10 mM. Protein extracts were obtained by cell lysis in RIPA buffer after suitable washes in PBS. Protein samples were separated onto SDS-PAGE and transferred to nitrocellulose membrane for immunoblotting. Monoclonal antibodies (mAb) used for the analyses were anti-LDHA (Cell Signalling Technologies, cat. #2012), anti-PKM (cat.#3198) and anti-GLUT-1 rabbit (cat.#12939). Beta-actin mouse mAb (cat. #3700) was used as a loading control. Goat Anti-Rabbit IgG-HRP Conjugate and Goat Anti-Mouse IgG-HRP Conjugate (BioRad, cat. #1706515 and cat.#1706516, respectively) were used as secondary antibodies. Primary antibodies were used at 1:1000 dilution, while secondary antibodies were used at 1:2000 dilution, according to the manufacturer's' instructions. The chemiluminescence reaction was performed by the WesternBright ECL HRP substrate kit (Advansta) and visualized by ChemiDoc MP Imaging system (BioRad). Band intensities were finally calculated by using Image Lab Software (BioRad).

Reference

1. Basso K, Liso A, Tiacci E, Benedetti R, Pulsoni A, Foa R, et al. Gene expression profiling of hairy cell leukemia reveals a phenotype related to memory B cells with altered expression of chemokine and adhesion receptors. J Exp Med. 2004 Jan 5;199(1):59-68.
2. Klein U, Tu Y, Stolovitzky GA, Keller JL, Haddad J, Jr., Miljkovic V, et al. Transcriptional analysis of the B cell germinal center reaction. Proc Natl Acad Sci U S A. 2003 Mar 4;100(5):2639-44.
3. Klein U, Tu Y, Stolovitzky GA, Mattioli M, Cattoretti G, Husson H, et al. Gene expression profiling of B cell chronic lymphocytic leukemia reveals a homogeneous phenotype related to memory B cells. J Exp Med. 2001 Dec 3;194(11):1625-38.
4. Piccaluga PP, Agostinelli C, Califano A, Rossi M, Basso K, Zupo S, et al. Gene expression analysis of peripheral T cell lymphoma, unspecified, reveals distinct profiles and new potential therapeutic targets. J Clin Invest. 2007 Mar;117(3):823-34.
5. Barretina J, Caponigro G, Stransky N, Venkatesan K, Margolin AA, Kim S, et al. The Cancer Cell Line Encyclopedia enables predictive modelling of anticancer drug sensitivity. Nature. 2012;483(7391):603-7.
6. Fitzsimmons L, Boyce AJ, Wei W, Chang C, Croom-Carter D, Tierney RJ, et al. Coordinated repression of BIM and PUMA by Epstein–Barr virus latent genes maintains the survival of Burkitt lymphoma cells. Cell Death & Differentiation. 2018;25(2):241-54.
7. Di Napoli A, De Cecco L, Piccaluga PP, Navari M, Cancila V, Cippitelli C, et al. Transcriptional analysis distinguishes breast implant-associated anaplastic large cell lymphoma from other peripheral T-cell lymphomas. Modern Pathology. 2019;32(2):216-30.
8. Navari M, Fuligni F, Laginestra MA, Etebari M, Ambrosio MR, Sapienza MR, et al. Molecular signature of Epstein Barr virus-positive Burkitt lymphoma and post-transplant lymphoproliferative disorder suggest different roles for Epstein Barr virus. Frontiers in microbiology. 2014;5:728.
9. Navari M, Etebari M, De Falco G, Ambrosio MR, Gibellini D, Leoncini L, et al. The presence of Epstein-Barr virus significantly impacts the transcriptional profile in immunodeficiency-associated Burkitt lymphoma. Frontiers in microbiology. 2015;6:556.
10. Irizarry RA, Bolstad BM, Collin F, Cope LM, Hobbs B, Speed TP. Summaries of Affymetrix GeneChip probe level data. Nucleic acids research. 2003;31(4):e15-e.
11. Johnson WE, Li C, Rabinovic A. Adjusting batch effects in microarray expression data using empirical Bayes methods. Biostatistics. 2007;8(1):118-27.
12. Subramanian A, Tamayo P, Mootha VK, Mukherjee S, Ebert BL, Gillette MA, et al. Gene set enrichment analysis: a knowledge-based approach for interpreting genome-wide expression profiles. Proc Natl Acad Sci U S A. 2005 Oct 25;102(43):15545-50.
13. Biagiotti S, Menotta M, Orazi S, Spapperi C, Brundu S, Fraternale A, et al. Dexamethasone improves redox state in ataxia telangiectasia cells by promoting an NRF2-mediated antioxidant response. The FEBS journal. 2016;283(21):3962-78.

2 Supplementary Tables

Supplementary Table 1. Glucose metabolism related genes differentially expressed in malignant lymphomas vs. non-neoplastic B-cells (T-test, $p<0.05$; fold change ≥2; Benjamini-Hockeberg False discovery rate)

**Gene Symbol**	**Entrez Gene**	**Corrected pvalue**	**Fold change**	**Regulation in disease**
FKBP4	2288	5,62E-08	2,10	up
ATP6V0C	527	2,71E-08	2,14	up
ATP1B1	481	4,86E-06	3,54	up
ATP1B1	481	1,64E-05	2,40	up
MGST3	4259	3,35E-15	2,65	up
TGFBI	7045	7,58E-23	6,77	up
IER3	8870	2,24E-09	3,61	up
IL13RA1	3597	1,79E-13	4,76	up
IL13RA1	3597	9,08E-09	3,01	up
DCN	1634	2,29E-07	5,48	up
G6PD	2539	7,74E-07	2,00	up
PAM	5066	0,003977623	2,24	up
MXI1	4601	1,31E-08	3,00	up
GNPDA1	10007	5,29E-22	3,11	up
PLOD2	5352	1,81E-06	3,34	up
SLC16A3	9123	7,19E-06	2,43	up
SDC3	9672	1,10E-11	3,03	up
PHYH	5264	5,20E-10	2,56	up
CD4	920	7,83E-08	2,75	up
CPT1A	1374	5,65E-11	2,23	up
ME1	4199	2,59E-05	2,07	up
COX7A1	1346	7,16E-08	2,11	up
VCAN	1462	1,54E-06	2,78	up
VCAN	1462	7,23E-09	4,17	up
BIK	638	0,026098564	2,08	down
MERTK	10461	3,94E-11	2,78	up
TXN	7295	1,21E-11	3,41	up
IRS2	8660	2,15E-04	2,09	up
IRS2	8660	2,76E-07	3,10	up
DCN	1634	1,37E-04	2,64	up
FBP1	2203	7,34E-05	2,70	up
IGFBP3	3486	2,55E-09	6,17	up

VEGFA	7422	7,60E-09	4,27	up
IL13RA1	3597	1,79E-06	2,41	up
IL13RA1	3597	1,57E-09	3,35	up
DCN	1634	5,98E-07	3,76	up
DCN	1634	1,95E-05	4,35	up
IGFBP3	3486	2,71E-06	2,98	up
SDC2	6383	1,02E-09	3,01	up
ZNF292	23036	3,97E-04	2,55	down
COL5A1	1289	1,28E-05	2,63	up
COL5A1	1289	8,46E-04	2,11	up
PAM	5066	0,00142284	2,03	up
VCAN	1462	2,32E-08	3,32	up
CYB5A	1528	6,76E-09	2,65	up
TXN	7295	4,67E-05	2,43	up
RRAGD	58528	3,78E-07	3,15	up
RRAGD	58528	7,79E-09	3,21	up
VCAN	1462	1,27E-08	4,40	up
SRD5A3	79644	1,62E-05	2,22	up
ND4	4538	4,51E-06	2,06	up
PDK4	5166	3,42E-09	3,56	up
AK4	205	5,63E-04	2,18	up
CD44	960	1,73E-12	2,37	down
ND2	4536	4,77E-09	2,32	up
ATP6	4508	5,60E-04	2,05	up
BPNT1	10380	7,01E-15	2,09	down

.

3 Supplementary Figures

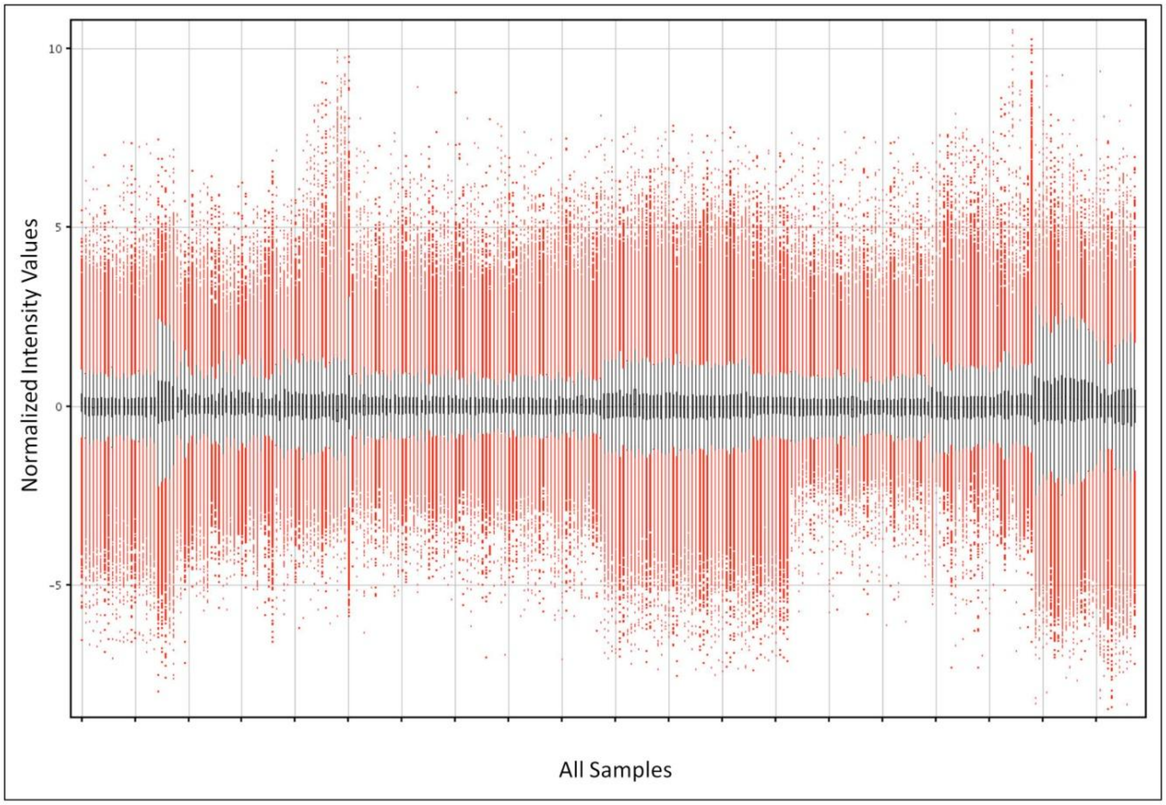

Supplementary Figure 1. *Data normalization.* Horizontal bar shows the samples and vertical bar indicates normalized intensity values.

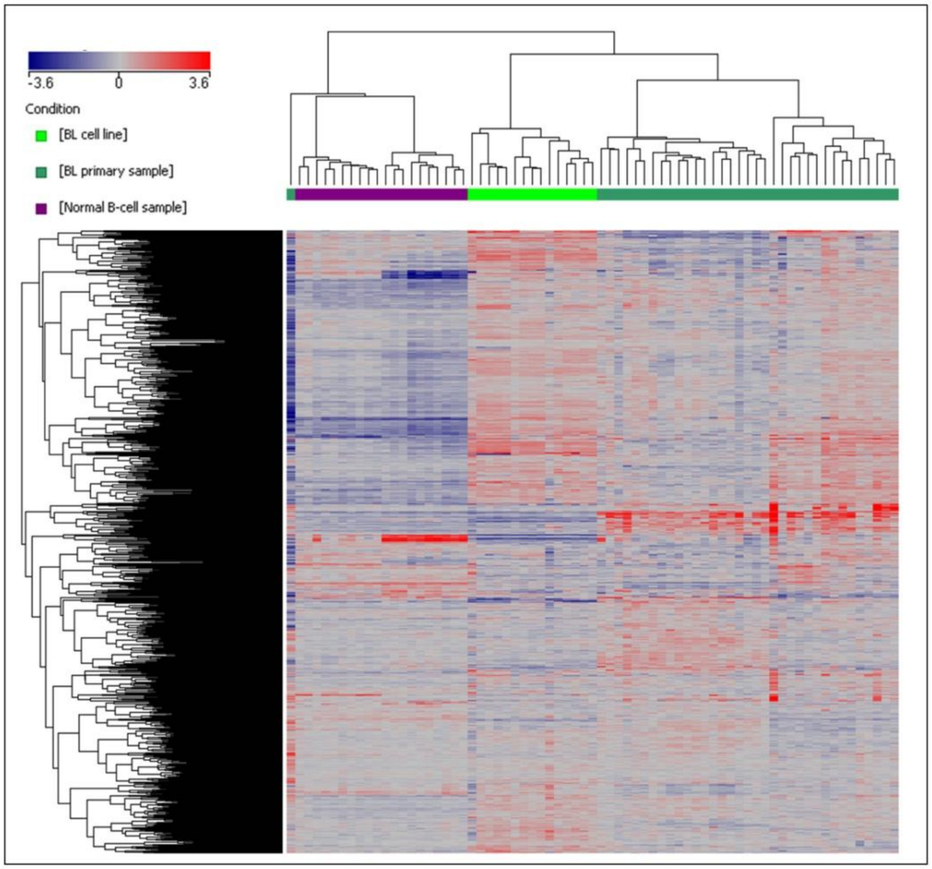

Supplementary Figure 2. *Hierarchical clustering of BL tumors and DAUDI cells and normal B-cells (PBL) based on the expression of glucose metabolism related genes.* Burkitt lymphoma primary samples (dark green), Burkitt lymphoma cell lines (light green) and normal B-cell samples (purple) are illustrates. All cell lines clustered with primary cases indicating that they can represent reliable models for studying Burkitt lymphoma glucose metabolism.

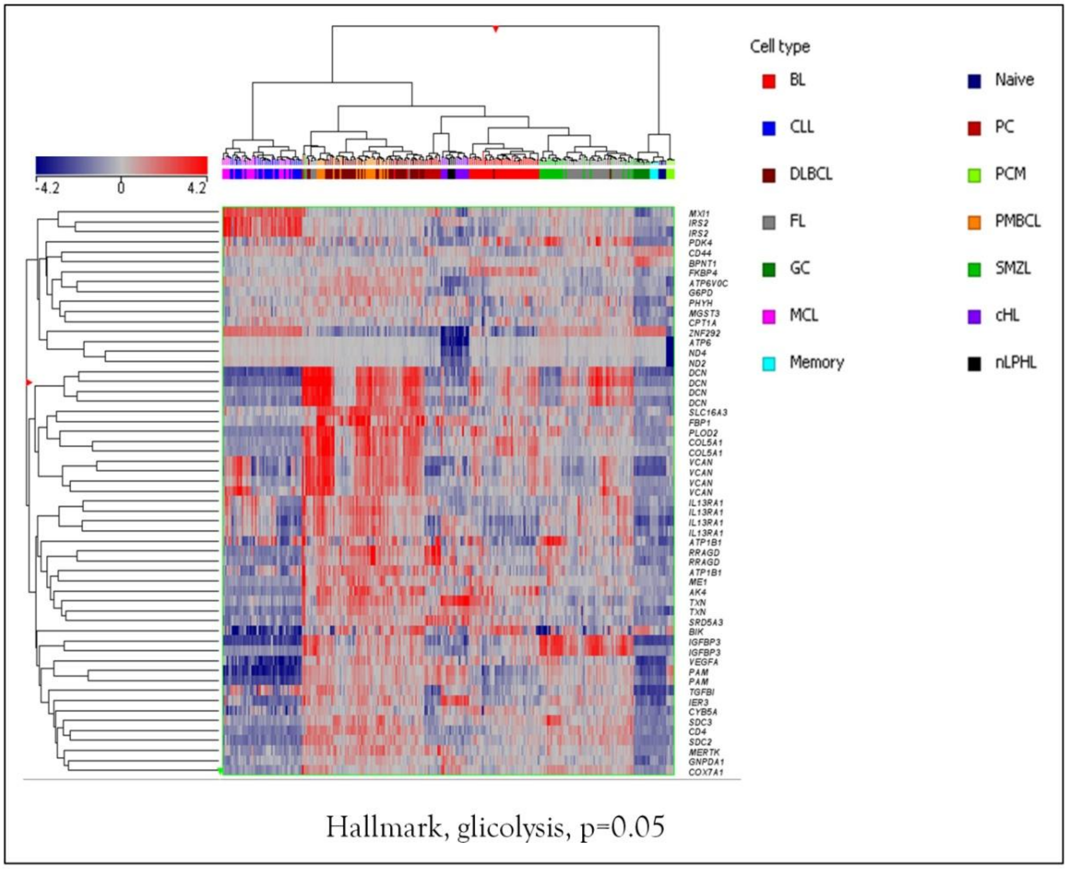

Supplementary Figure 3. Hierarchical clustering of human malignant lymphomas vs. normal B-cell subsets based on the expression of glucose metabolism associtaed genes.

In the matrix, each column represents a sample and each row represents a gene. The color scale bar shows the relative gene expression changes normalized by the standard deviation (0 is the mean expression level of a given gene).

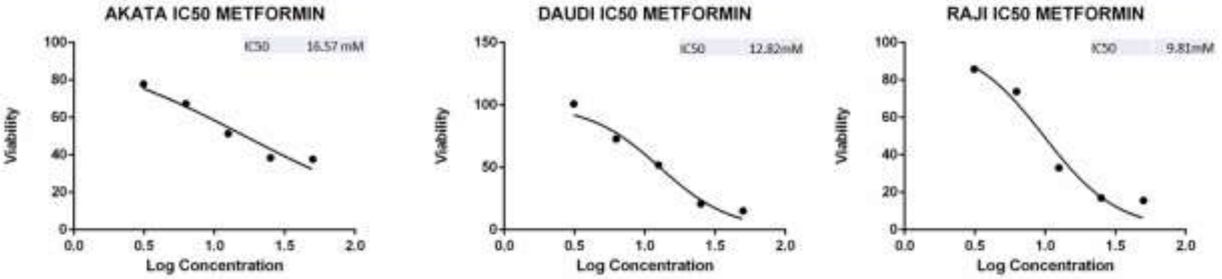

Supplementary Figure 4. MTS analysis of DAUDI, AKATA, and RAJI cell lines treated with metformin. Cell viability was assessed after 48 hours of exposure to the drug (0, 3.125 mM, 6.25 mM, 12.5 mM, 25 mM, 50 mM). IC50 was calculated in GraphPad (https://www.graphpad.com/).

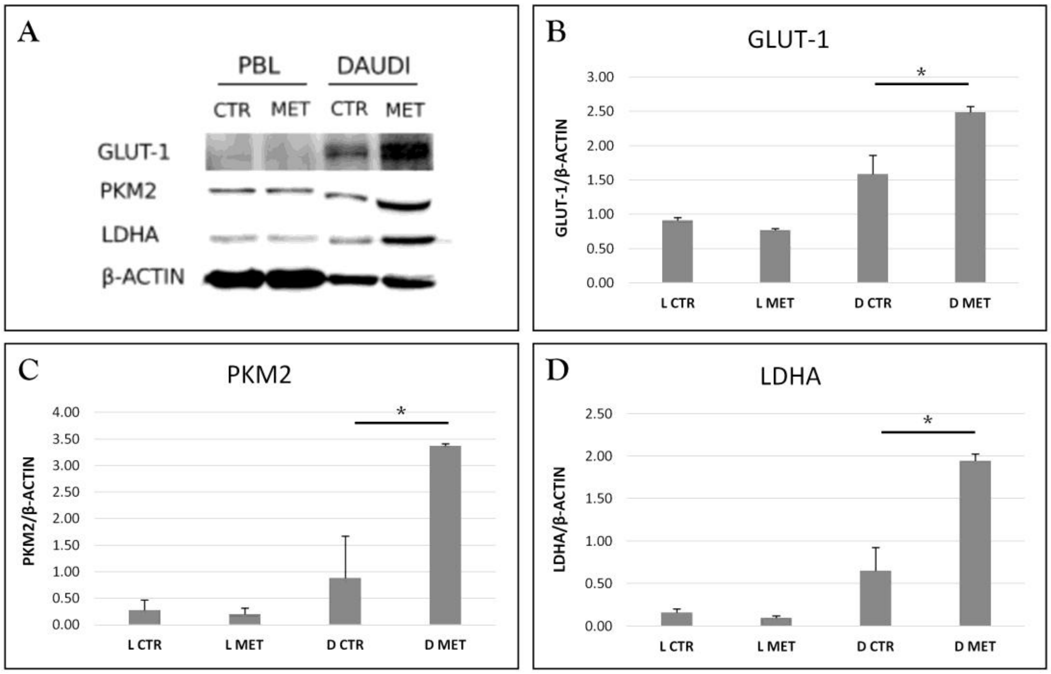

Supplementary Figure 5. *Western blotting evaluation of protein levels of GLUT-1, PKM2* and *LDHA in DAUDI and PBL cells treated with metformin*. After 24 hours of 10 mM metformin treatment, protein levels were assessed by western blotting on total protein extracts from the cells. Beta-actin was used as the loading control. A represents an example of membrane development, while B, C and D indicate the comparison of band intensities for *GLUT-1, PKM2* and *LDHA,* respectively. CTR: untreated cells; MET: treated cells with 10mM metformin. Values are means and SEM of four independent experiments (Wilcoxon signed rank test; *two-tailed p-values≤0.05).

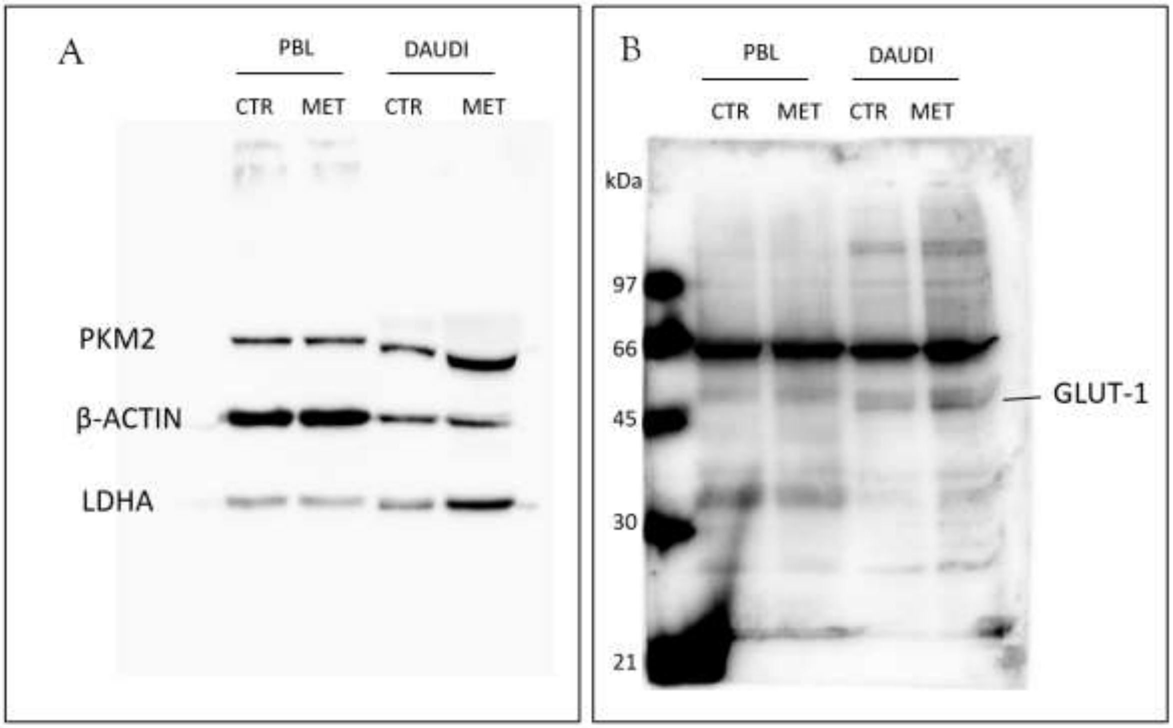

Supplementary Figure 6. *Representative full-lenght blots used for the quantification of GLUT-1, PKM2 and LDHA protein levels in DAUDI and PBL cells treated with metformin*. A) On the same membrane we performed a co-hybridization of anti-PKM2, anti-LDHA and anti-β-actin (loading control) antibodies. B) We performed the hybridization of anti-GLUT-1 antibody in a separated membrane to avoid any interference due to the others antibodies used. The bands above those of GLUT-1 represent anti-PKM2 antibody hybridization; we cannot use anti-β-actin on the same membrane because the signal would have maskered that of GLUT-1. CTR: untreated cells; MET: treated cells with 10mM metformin.
